# Molecularly Imprinted Electrochemical Sensor Based on Palladium@Yttrium Oxide@Boronnitride Nanocomposite for Determination of Glyphosate Herbicide in Drinking Water Samples

**DOI:** 10.3390/foods15010007

**Published:** 2025-12-19

**Authors:** Bahar Bankoğlu Yola, Sena Bekerecioğlu, İlknur Polat, Ülkü Melike Alptekin, Necip Atar, Mehmet Lütfi Yola

**Affiliations:** 1 Department of Engineering Basic Sciences, Faculty of Engineering and Natural Sciences, Gaziantep Islam Science and Technology University, Gaziantep 27260, Türkiye; bahar.bankogluyola@gibtu.edu.tr; 2 Department of Nutrition and Dietetics, Faculty of Health Sciences, Hasan Kalyoncu University, Gaziantep 27010, Türkiye; sena.bekerecioglu@hku.edu.tr (S.B.); ilknur.polat@hku.edu.tr (İ.P.); 3 Department of Medical Services and Techniques, Dörtyol Vocational School of Health Services, Iskenderun Technical University, Hatay 31200, Türkiye; ulku.alptekin@iste.edu.tr; 4 Department of Chemical Engineering, Faculty of Engineering, Pamukkale University, Denizli 20160, Türkiye; natar@pau.edu.tr; 5 Department of Biology, Faculty of Science, Ankara University, Ankara 06100, Türkiye

**Keywords:** glyphosate, nanocomposite, molecularly imprinting polymer, voltammetry, drinking water

## Abstract

Glyphosate (GLY) is a systemic herbicide used in agriculture and has a carcinogenic effect after long-term usage. Herein, a molecularly imprinted electrochemical sensor based on palladium@yttrium oxide@boron nitride nanosheets (Pd/Y_2_O_3_@BN) nanocomposite was developed for the detection of GLY in drinking water. After the preparation of Pd/Y_2_O_3_@BN nanocomposite by using sonication and NaBH_4_ reduction methods, Pd/Y_2_O_3_@BN nanocomposite as electrode material was applied on glassy carbon electrode by infrared lamp. Then, a molecularly imprinted glassy carbon electrode based on Pd/Y_2_O_3_@BN (MIP) was designed with cyclic voltammetry (CV) in presence of pyrrole monomer and GLY molecule. After the spectroscopic and microscopic characterizations, the linearity in the range of 1.0 × 10^−9^–1.0 × 10^−8^ M with a detection limit (LOD) of 3.3 × 10^−10^ M was obtained for GLY molecule. After MIP electrode was applied to drinking water samples with high recovery, the selectivity, stability, repeatability, and reproducibility features were studied. These promising results suggested that the as-fabricated MIP electrode presented a novel and highly effective approach for GLY assay.

## 1. Introduction

Herbicides are chemicals used in agriculture and landscaping to destroy or control unwanted plants, or weeds. They cause plant death by inhibiting the biological processes involved in plant growth and development [1]. Among these chemicals, GLY, which has herbicidal activity, is a non-selective organophosphate pesticide and is effective against weeds. In addition to the development of GLY-resistant transgenic crops such as cotton and canola in agriculture against weeds [2], it is also frequently used in forestry, urban areas, orchards, home gardens, parks and, in recent years, in waterways [3]. Although this biochemical process does not take place in humans or animals, the occurrence of this process causes the target plants to stop growing and die over time [4,5]. Although GLY is said to have minimal side effects and considered safer than many herbicides, it has been stated that it may have negative effects on both the environment and human health with its recent increase in use [6]. With its wide range of uses, it is used extensively in many areas, especially in agriculture [2] and accumulates in both surface and underground areas [7]. The World Health Organization (WHO) designated GLY as “probably carcinogenic to humans” in 2015 [8]. However, USEPA has classified GLY as “non-carcinogenic” to humans in Group E and the European Food Safety Authority (EFSA), claims that it is a potent carcinogen [9,10]. In a study, it was detected in human urine, revealing the biological activity and potential health risks of this chemical [11,12]. In addition to the controversial cancer risk of GLY [13], it has negative effects on the immune system [14], nervous system [15], cardiovascular system [16], and respiratory system [17]. Although GLY residues in the studies do not exceed the threshold values, it is stated that its negative effects cannot be ignored [18,19]. In the literature, chromatographic and immunochemical-based methods such as HPLC, ion chromatography (IC), gas chromatography and ELISA were frequently used for GLY assay [20,21,22,23]. These methods are difficult to implement due to their high cost, lengthy processing steps, and the need for trained personnel. However, more sensitive, rapid, and reliable methods for detecting GLY have recently become popular [24]. In addition, electrochemical methods are based on the measurement of electrical signals occurring at the electrode surface. Among electrochemical methods, voltammetry can offer some advantages such as low cost, rapidity, and field applicability, and it is frequently used today in food, environmental, pharmaceutical, and biosensor applications [25,26,27].

The metal based nanoparticles and two-dimensional materials have been intensively studied to make better the electrochemical sensor performance [28,29,30,31]. Especially, palladium nanoparticle-based catalysts have been reported for electrochemical sensor/biosensor applications [32,33]. In addition, the rare earth metals including cerium, niobium and yttrium can increase the nanomaterial’s chemical/physical properties and sensor performance. Y_2_O_3_-based nanocomposites were investigated in terms of the catalytic conversion belonging to organic compounds. However, their electrochemical properties have not been elucidated in detail yet [34,35]. Furthermore, two-dimensional materials including graphene [36,37] and boronnitride have been frequently used as modified electrode materials in electrochemical sensor applications in recent years [38,39]. In particular, these nanomaterials can enhance the metal dispersion, electrochemical active surface area, and the effective electron transfer in the process sensor applications [40,41]. Boronnitride nanomaterials have been extensively investigated owing to the superior features such as easy functionalization with the other nanomaterials and metals, high affinity, sensor applications, and carrier mobility. In addition, the large band gap between the layers of boronnitride nanosheets can make it transparent on the visible region. Moreover, because of conductivity and effective surface area properties, these nanomaterials have significant electrochemical sensor applications. For example, polydopamine@hexagonal boronnitride nanomaterials were utilized for electrochemical detection of T-Tau as a marker of Alzheimer’s disease [42]. In addition, gold nanoparticles@boronnitride and palladium nanoparticles@boronnitride nanocomposites have been used for pesticide analysis such as carbendazin and aflatoxin B1, respectively [43,44].

Molecularly imprinted polymers (MIPs) are notable not only for their high selectivity but also for their robust mechanical structure [45,46,47]. Their resistance to high temperatures and pressures, as well as their ability to maintain their functionality even in aggressive environments containing acidic, basic, metal ions, or organic solvents, make them ideal for demanding applications [48]. MIPs can also be used as synthetic recognition elements in various applications due to their high stability and compatibility of polymerization steps with sensor technologies [49]. The method basically consists of three main steps: Pre-complex formation, polymerization, and the target molecule removal [50]. The development of MIP-based sensors for many target substances such as pesticides has been reported [51].

This study demonstrated a new perspective for instantaneous and selective analysis of herbicide-type pesticides based on Pd/Y_2_O_3_@BN nanocomposite and MIPs. Hence, molecularly imprinted electrochemical electrodes based on Pd/Y_2_O_3_@BN nanocomposite were designed and applied to glyphosate detection in drinking water samples for the first time in the literature. Thus, thanks to the created molecularly imprinting sensor, it will be possible to estimate crucial health problems resulting from pesticide exposure.

## 2. Materials and Methods

### 2.1. Materials

GLY, carbendazim (CAR), mesotrione (MES), irgasan (IRG), yttrium nitrate (Y_2_NO_3_), boron nitride powder (purity of 99.0%), sodium borohydride (NaBH_4_), poly(diallyldimethyl ammonium chloride) solution (PDDA), potassium palladium (II) tetrachloride (K_2_PdCl_4_) and pyrrole (Py) monomer were purchased from Sigma-Aldrich Merck Group (Darmstadt, Germany). Phosphate-buffered saline (pH 5.0, 0.1 mol L^−1^ PBS) was selected as a supporting electrolyte.

### 2.2. Instrumentation

The used apparatus for this study was presented in Supplementary Materials.

### 2.3. Synthesis of Boron Nitride Nanosheets and Pd/Y_2_O_3_@BN Nanocomposite

The boron nitride nanosheets (BN) were prepared by sonication method. For this aim, boron nitride powder of 1.0 g was dispersed in pure water (1.0 L) and sonicated for 5 h at 25 °C. Then, the centrifugation treatment was performed at 10,000 rpm for 1 h and washed with pure water [52]. After that, the synthesis of PDDA-incorporated BN was carried out (PDDA@BN). After the preparation of dispersion including BN (0.50 g) in pure water (300.0 mL), the solution was mixed with PDDA (1.0 mL, 15.0 wt % in pure water) and the suspension was exposed to sonication for 6 h at 25 °C. After the centrifugation treatment at 10,000 rpm for 1 h, the solid was washed four times with pure water and the precipitate was dried at 75 °C.

Pd/Y_2_O_3_@BN nanocomposite was synthesized by NaBH_4_ reduction. PDDA@BN (0.50 g) was dispersed in pure water (300.0 mL) and the suspension was sonicated for 30 min at 25 °C [53]. K_2_PdCl_4_ (3.0 mL, 6.0 mg mL^−1^) was slowly dripped and Y_2_NO_3_ solution (1.0 mL, 2.0 mg mL^−1^) was added into this dispersion. Then, NaBH_4_ solution (10.0 mL, 2.0 mg mL^−1^) was slowly dripped until color transformation was observed. Finally, the dispersion was centrifuged at 10,000 rpm for 30 min and the product was dried at 100 °C during 90 min. Likewise, Y_2_O_3_@BN nanocomposite was synthesized by NaBH_4_ reduction without K_2_PdCl_4_ (3.0 mL, 6.0 mg/mL).

### 2.4. Preparation of Pd/Y_2_O_3_@BN Modified Glassy Carbon Electrode (Pd/Y_2_O_3_@BN/GCE)

The polishing method of GCE was presented in our previous work [54]. Then, the aqueous Pd/Y_2_O_3_@BN nanocomposite suspension (20.0 μL, 0.20 mg mL^−1^) was dropped on GCE and dried under IR lamp (Pd/Y_2_O_3_@BN/GCE). For comparative electrochemical results, the other modified GCE surfaces (Y_2_O_3_@BN/GCE and BN/GCE) were developed by using the same procedure.

### 2.5. Development of GLY Imprinted Electrode and GLY Removal

**Scheme 1:**
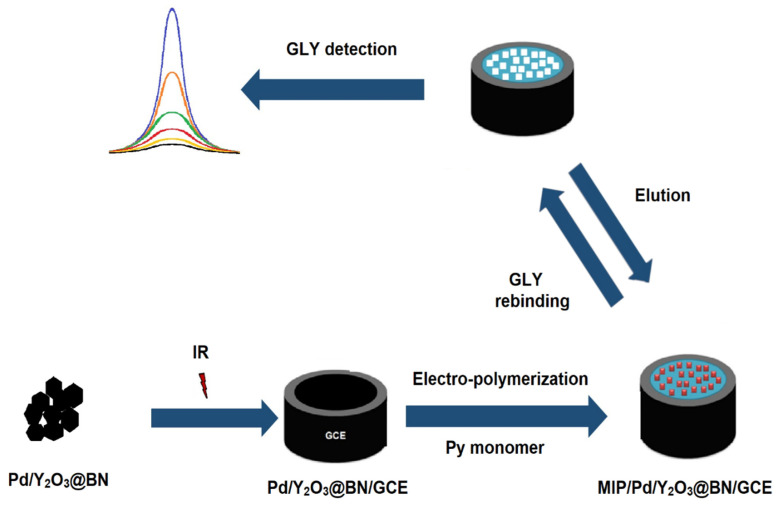
Schematic demonstration of MIP/Pd/Y_2_O_3_@BN/GCE.

Firstly, the suspension (pH 5.0) including 25.0 mmol L^−1^ GLY and 100.0 mmol L^−1^ Py monomer was prepared in pure water and transported to the electrochemical cell. After the passing of nitrogen gas into the electrochemical cell to eliminate the dissolved oxygen for 20 min, the monitoring process at approximately +0.70 V in 0.0/+1.0 V potential range relating to GLY:monomer polymerization was performed by the CV method. GLY imprinted polymeric cavities were created on the electrode surface by completing 25 CV scanning cycles. After the washing treatment by pure water four times, the formed electrodes were stored at 25 °C (MIP/Pd/Y_2_O_3_@BN/GCE). To demonstrate the provided superior selectivity by the molecular imprinting technique, GLY non-imprinted electrodes (NIP) were prepared using the same technical procedure without the GLY molecule. Scheme 1 revealed the development of MIP/Pd/Y_2_O_3_@BN/GCE.

For GLY removal from the electrode surface, the electrostatic/hydrogen bond interactions between target and the monomer were overcome by using 0.1 mol L^−1^ NaCl as the desorption solution. For this aim, MIP electrodes were firstly put in a conical flask containing the desorption solution and shaken for 20 min. After that, MIP electrodes were dried at 25 °C.

### 2.6. Sample Preparation

The drinking water was purchased from the supermarket in Ankara, Turkey. After the drinking water (20.0 mL) was transported into volumetric flask (50.0 mL), the centrifugation was performed to remove solid residues for 10 min. Finally, the sample was diluted with pure water to fall within the linearity range.

## 3. Results and Discussion

### 3.1. Characterizations of Pd/Y_2_O_3_@BN

**Figure 1:**
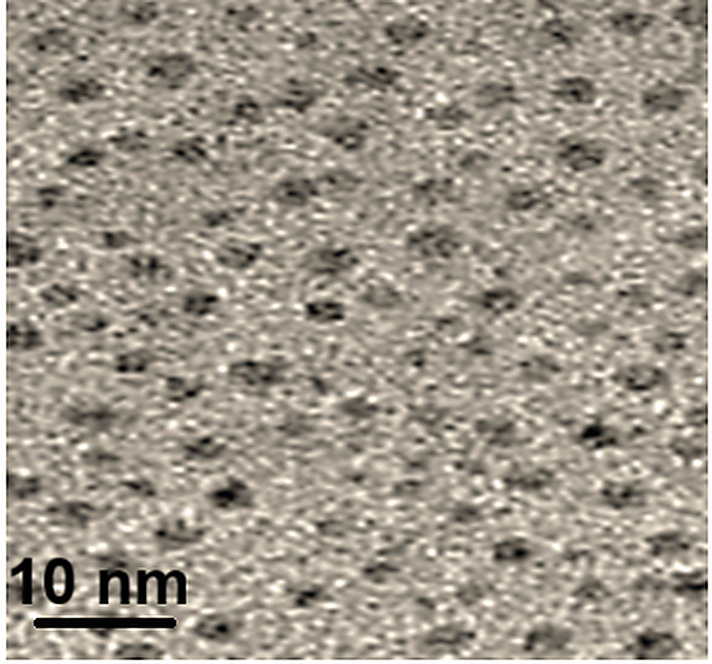
TEM image of Pd/Y_2_O_3_@BN nanocomposite.

Figure 1 depicted TEM image of Pd/Y_2_O_3_@BN nanocomposite. According to Figure 1, the crystalline and uniform distribution of Pd/Y_2_O_3_ nanoparticles on boron nitride nanosheets had an average size of 5–9 nm. In addition, according to EDX MAPS spectrum (Figure S1), the presence of palladium, yttrium, oxygen, boron, and nitrogen indicated the successful preparation of Pd/Y_2_O_3_@BN nanocomposite.

**Figure 2:**
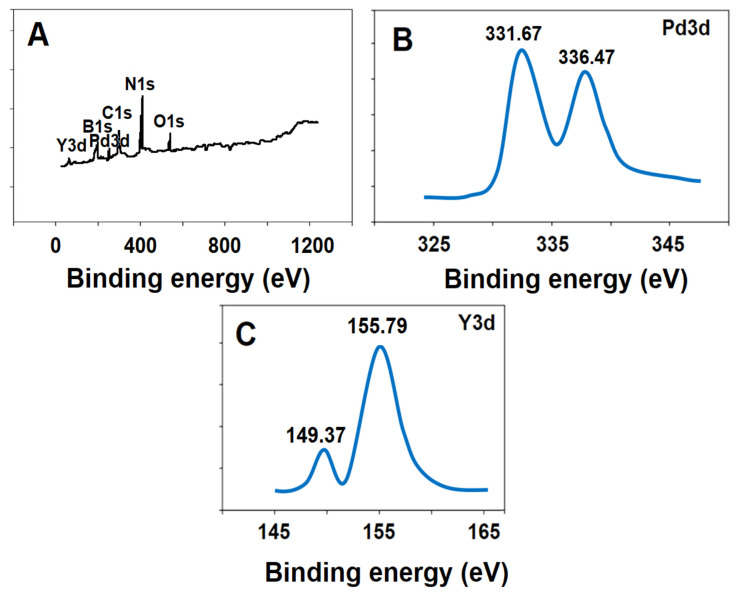
(**A**) XPS survey spectra of Pd/Y_2_O_3_@BN nanocomposite, (**B**) XPS core level of Pd3d and (**C**) XPS core level of Y3d.

XPS measurements of Pd/Y_2_O_3_@BN nanocomposite were investigated for the elemental analysis of the nanocomposite. According to Figure 2A, the presence of boron, nitrogen, carbon, oxygen, palladium, and yttrium indicated the formation of Pd/Y_2_O_3_@BN nanocomposite [55]. XPS spectrum of palladium was attributed to two XPS peaks at 336.47 eV and 331.67 eV owing to 3D electrons (Figure 2B) [56]. XPS peak at 155.79 eV and 149.37 eV also corresponded to yttrium (Figure 2C).

Furthermore, XRD results were obtained to confirm the presence of Pd/Y_2_O_3_@BN nanocomposite. According to Figure S2A, XRD peaks at 26.47°, 38.17°, 43.89°, and 54.17° corresponded to the (002), (100), (101), (102), and (104) plane for hexagonal boronnitride material. In addition, XRD peaks at 40.09° corresponded to the presence of palladium [57], and XRD peaks at 43.67° and 57.43° were attributed to yttrium oxide (Figure S2B) [53].

### 3.2. Electrochemical Studies of Boronnitride Nanosheets, Y_2_O_3_@BN and Pd/Y_2_O_3_@BN Modified Electrodes

**Figure 3:**
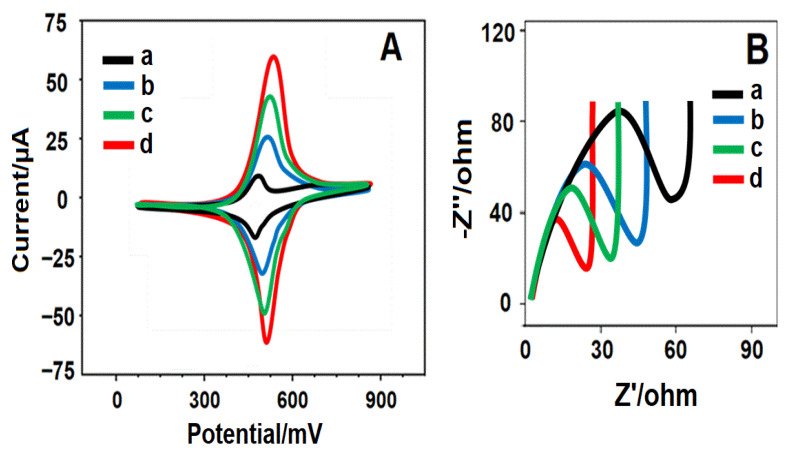
(**A**) CV curves and (**B**) EIS responses at (a) bare GCE, (b) BN/GCE, (c) Y_2_O_3_@BN/GCE, (d) Pd/Y_2_O_3_@BN/GCE (Potential scan rate: 100 mV s^−1^).

The electrochemical behaviors of boronnitride nanosheets, Y_2_O_3_@BN and Pd/Y_2_O_3_@BN nanocomposite were evaluated by CV (Figure 3A) and EIS (Figure 3B) methods in the presence of 1.0 mmol L^−1^ [Fe(CN)_6_]^3−/4-^ containing 0.1 mol L^−1^ KCl. The bare GCE revealed obvious redox peak with peak potential separation of ∆Ep = 30 mV. After that, an important increase was observed in redox peak currents with peak potential separation of ∆Ep = 40 mV on BN/GCE owing to the increased surface area of boronnitride material [58]. It was found that larger anodic and cathodic peak currents on Y_2_O_3_@BN/GCE in comparison with BN/GCE were observed due to the chemical activity of Y_2_O_3_. Thus, Y_2_O_3_ improved the catalytic behavior of boronnitride nanosheets. Lastly, Pd/Y_2_O_3_@BN/GCE demonstrated the highest peak current with peak potential separation of ∆Ep = 50 mV owing to the synergistic effect of boronnitride nanosheets, Y_2_O_3_ and palladium. The electroactive surface areas of bare GCE, BN/GCE, Y_2_O_3_@BN/GCE and Pd/Y_2_O_3_@BN/GCE were calculated as 0.073 ± 0.002, 0.349 ± 0.003, 0.519 ± 0.001, and 0.839 ± 0.003 cm^2^ by using i_p_ = 2.69 × 10^5^ A n^3/2^ D^1/2^ C v^1/2^ equation in the presence of 1.0 mmol L^−1^ [Fe(CN)_6_]^3−^, respectively. In conclusion, the high conductivity of boronnitride materials and the superior catalytic performance of Y_2_O_3_ and palladium provided the important synergistic effect. More importantly, the modification process enhanced the electrode surface area, providing a higher current response. According to EIS results, the charge transfer resistance (R_ct_) values were evaluated to be 60 ohm for bare GCE (curve a), 45 ohm for BN/GCE (curve b), 30 ohm for Y_2_O_3_@BN/GCE (curve c) and 20 ohm for Pd/Y_2_O_3_@BN/GCE (curve d).

To confirm electrochemical mechanism including diffusion, a linear dependency was observed between currents and the square root of the scan rate (10–1000 mV s^−1^), verifying electrochemical mechanism with diffusion. The linear equations of Ipa = 10.4973(v)^1/2^ + 7.2793 (R^2^ = 0.9998) and Ipc = − 10.10737(v)^1/2^ − 6.3749 (R^2^ = 0.9997) were obtained.

In addition, the peak potentials belonging to anodic and cathodic observations were shifted to positive and negative values with an increase in the scan rate, indicating reversible electron transfer on Pd/Y_2_O_3_@BN/GCE.

### 3.3. Development of GLY Imprinted Polymer on Pd/Y_2_O_3_@BN/GCE

**Figure 4:**
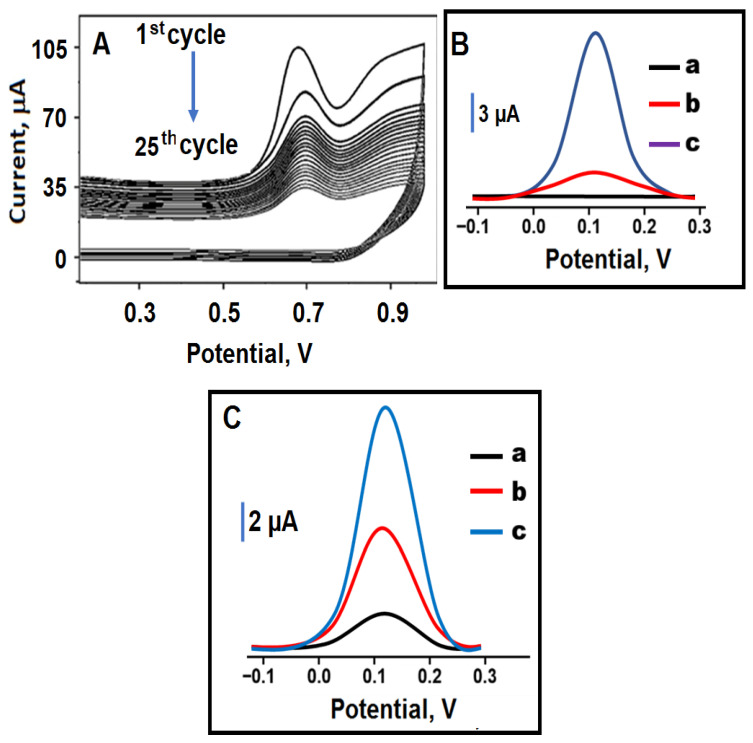
(**A**) Electro-polymerization voltammograms on Pd/Y_2_O_3_@BN/GCE (Scan rate: 100 mV s^−1^); (**B**) SWVs of the prepared electrodes: (a) MIP/Pd/Y_2_O_3_@BN/GCE in blank buffer solution (pH 5.0), (b) NIP/Pd/Y_2_O_3_@BN/GCE after rebinding of 10.0 nmol L^−1^ GLY, (c) MIP/Pd/Y_2_O_3_@BN/GCE after rebinding of 10.0 nmol L^−1^ GLY; (**C**) SWVs of different molecularly imprinting electrodes after rebinding of 10.0 nmol L^−1^ GLY (a) MIP/bare GCE, (b) MIP/BN/GCE, and (c) MIP/Pd/Y_2_O_3_@BN/GCE (frequency of 50 Hz, pulse amplitude of 20 mV, scan increment of 3 mV for SWV measurements).

Figure 4A demonstrated the electro-polymerization voltammograms in 100.0 mmol L^−1^ Py monomer and 25.0 mmol L^−1^ GLY on Pd/Y_2_O_3_@BN/GCE in a potential between +0.0/+1.0 V in the presence of 0.1 mol L^−1^ PBS (pH 5.0). The observed peak at approximately +0.70 V with the applied high potential indicated the start of polymerization. According to scan rate of 100 mV s^−1^, 25 CV scans took approximately 500 s (at least 8.3 min of enrichment time) to complete. The current signal decreases with the number of scans indicated the formation of GLY imprinted polymeric nano-cavities on Pd/Y_2_O_3_@BN/GCE. It was observed that this peak signal approached zero with the 25th scan. This situation revealed the formation of molecularly imprinting polymer layers on Pd/Y_2_O_3_@BN/GCE.

To prove the high selectivity of molecular imprinting technique in sensor technology applications, the prepared MIP and NIP electrodes were separately activated in the presence of 10.0 nmol L^−1^ GLY in 0.1 mol L^−1^ PBS (pH 5.0). The current values for MIP/Pd/Y_2_O_3_@BN/GCE and NIP/Pd/Y_2_O_3_@BN/GCE were calculated as 14.04 and 1.75 µA, respectively. The current value of MIP/Pd/Y_2_O_3_@BN/GCE was 8.02 times higher than that of NIP/Pd/Y_2_O_3_@BN/GCE, providing high selectivity in GLY detection. In addition, the obtained small signal current using the NIP electrode was due to nonspecific interactions occurring at the surface (Figure 4B). In addition, SEM images showed the morphological differences between the MIP and NIP electrodes. More porous surfaces were recorded on MIP electrode (Figure S3A) and non-porous polymeric layers were obtained on NIP electrode (Figure S3B).

Finally, Figure 4 showed that the electrochemical behaviors of various MIP electrodes such as MIP/bare GCE, MIP/BN/GCE and MIP/Pd/Y_2_O_3_@BN/GCE were investigated in the presence of 10.0 nmol L^−1^ GLY in 0.1 mol L^−1^ PBS (pH 5.0). Thus, by combining the synergistic effect and high conductivity properties of the Pd/Y_2_O_3_@BN nanocomposite with the high selectivity properties of MIPs, a novel MIP/Pd/Y_2_O_3_@BN/GCE electrode with high catalytic effect for GLY was successfully prepared.

### 3.4. Optimization

#### 3.4.1. pH Effect

GLY as a potent chelating agent can form different complexes with Mg, Zn, and Ca on pH dependency [59]. At acidic pH values, GLY converted to anionic form owing to the ionization of carbonyl and phosphate groups, and this situation gained the increase in affinity between monomer and GLY analyte [60]. To determine the optimum pH value, the medium pH was prepared between pH 4.0–9.0 and electrochemical signal values were recorded in the presence of 10.0 nmol L^−1^ GLY. According to Figure S4A, it was observed that the highest affinity interaction between GLY and the monomer occurred at pH 5.0 medium and the optimum pH was selected as 5.0 [61].

#### 3.4.2. Mole Ratio GLY to Py Monomer Effect

In molecularly imprinted electrochemical sensor studies, the stoichiometric nature of the formed pre-complex between the target molecule and the monomer significantly affects the sensor sensitivity. In particular, the amount of monomer interacting with the target molecule must be carefully optimized. In cases where the monomer ratio was lower than 100.0 mmol L^−1^, the specific GLY:Py interaction on the electrode surface could be less, and therefore the GLY-specific polymeric cavities on the electrode surface could be less. Conversely, if the monomer ratio was higher than 100.0 mmol L^−1^, nonspecific interactions may occur between the monomer and the nanocomposite material on the electrode surface. Hence, GLY imprinted electrodes were prepared by using 100.0 mmol L^−1^ Py monomer and 25.0 mmol L^−1^ GLY, showing the highest electrochemical signal (Figure S4B).

#### 3.4.3. Elution Time Effect

Another important factor was elution time. If the target molecule was effectively removed from the electrode surface, the higher sensor sensitivity was observed. A significant increase in electrochemical signals was observed up to 20 min of elution time. The stabilization of the signals after 20 min proved that GLY molecules were completely removed from MIP/Pd/Y_2_O_3_@BN/GCE. Thus, the elution time of 20 min was selected as optimum desorption time (Figure S4C).

#### 3.4.4. Scan Cycle Effect

In MIP sensor applications, scan cycle values should be optimized since a thick polymeric layer on the electrode surface can prevent the removal target molecule from the electrode surface. In the opposite case, sensor sensitivity may be negatively affected because a thin polymeric surface can break away from the electrode surface. Several scan cycles in the range from 15 to 55 were investigated on currents. In conclusion, the scan cycle number of 25 was selected as optimum scan cycle (Figure S4D).

### 3.5. Sensitivity of MIP/Pd/Y_2_O_3_@BN/GCE Electrode and Recovery Studies

**Figure 5:**
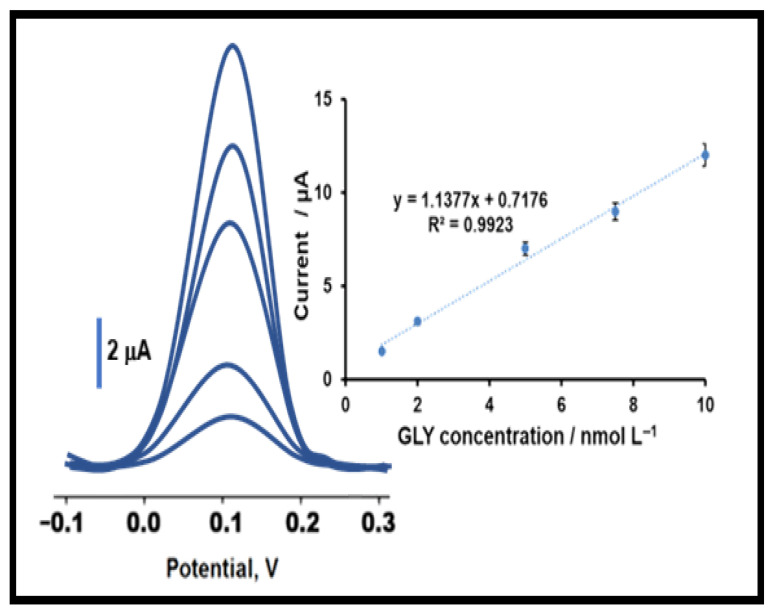
GLY concentration effect on MIP/Pd/Y_2_O_3_@BN/GCE electrode in presence of 0.1 mol L^−1^ PBS (pH 5.0) (from 1.0 × 10^−9^ to 1.0 × 10^−8^ mol L^−1^ GLY) by SWV method. Inset: Calibration curve of GLY (frequency of 50 Hz, pulse amplitude of 20 mV, scan increment of 3 mV for SWV measurements).

The SWVs responses for the prepared MIP/Pd/Y_2_O_3_@BN/GCE electrode under optimal conditions, including pH 5.0, the desorption time of 20 min, and the scan cycle number of 25 and (1:4) mole ratio, for different amounts of GLY were recorded for the investigation of the sensor’s analytical performance. The results revealed that the current signals on MIP/Pd/Y_2_O_3_@BN/GCE enhanced with GLY concentrations (Figure 5). The linear relationship between amounts of GLY vs. SWV peak current was observed in calibration plot with I (µA) = 1.1377 C_GLY_ (nmol L^−1^) + 0.7176 (the inset of Figure 5). The presented MIP sensor showed sensitive working range from 1.0 × 10^−9^ to 1.0 × 10^−8^ mol L^−1^ GLY. The limit of quantification (LOQ), and LOD values were obtained as 1.0 × 10^−9^ mol L^−1^ and 3.3 × 10^−10^ mol L^−1^ in drinking water, respectively (see Supplementary Materials for the equations), demonstrating the successful preparation of a highly sensitive analytical sensor for GLY determination. In addition, Table 1 revealed the comparison of GLY imprinted Pd/Y_2_O_3_@BN/GCE’s analytical performance with the other methods. The advantages of Pd/Y_2_O_3_@BN nanocomposite in fabricating the electrochemical sensor were as follows: (i) It was possible to say that the most sensitive sensor system among the prepared analytical methods for the determination of GLY was presented in the literature. (ii) A sensor preparation protocol close to zero waste was implemented thanks to the sonication and simple reduction methods when synthesizing the Pd/Y_2_O_3_@BN nanocomposite during the sensor preparation process. For this reason, a sensor system, which was environmentally friendly, harmless to human health, and easy to use has been presented for pesticide detections. (iii) This prepared sensor showed the high potential of Pd/Y_2_O_3_@BN nanocomposites as low-cost materials for electrochemical sensor applications. Subsequently, early diagnosis of possible health problems that may occur in case of exposure to this type of herbicides will be possible thanks to this study.

To prove the validity of the prepared sensor, MIP/Pd/Y_2_O_3_@BN/GCE electrode was conducted to real drinking water samples. According to Table 2, nearly 100.0% of the recovery results revealed that MIP/Pd/Y_2_O_3_@BN/GCE could carry out a GLY analysis from drinking water samples with high selectivity. Also, as another way to prove the validity of the MIP/Pd/Y_2_O_3_@BN/GCE electrode, the recovery values were compared by performing GLY analysis with another ICP/MS method available in the literature [68]. The absence of a significant difference between the results showed high accuracy of MIP/Pd/Y_2_O_3_@BN/GCE electrode.

To verify high selectivity of MIP/Pd/Y_2_O_3_@BN/GCE electrode, the standard addition method [(I (µA) = 1.1306 C_GLY_ (nmol L^−1^) + 1.467] was conducted to drinking water samples, and the slopes of the direct calibration and standard addition techniques were found almost nearby. This situation showed that all competing organic/inorganic agents present in the drinking water samples did not affect the analysis results, verifying the high reliability and selectivity of the prepared MIP/Pd/Y_2_O_3_@BN/GCE electrode.

### 3.6. Selectivity, Stability, Repeatability and Reproducibility of MIP/Pd/Y_2_O_3_@BN/GCE Electrode

**Figure 6:**
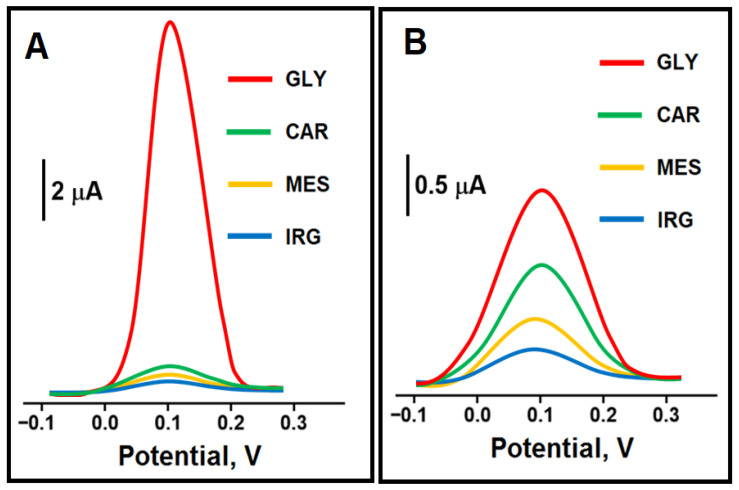
SWVs of (**A**) MIP/Pd/Y_2_O_3_@BN/GCE and (**B**) NIP/Pd/Y_2_O_3_@BN/GCE in 10.0 nmol L^−1^ GLY, 100.0 nmol L^−1^ CAR, 100.0 nmol L^−1^ MES and 100.0 nmol L^−1^ IRG in 0.1 mol L^−1^ PBS (pH 5.0) (frequency of 50 Hz, pulse amplitude of 20 mV, scan increment of 3 mV for SWV measurements).

To demonstrate the high selectivity of the prepared MIP/Pd/Y_2_O_3_@BN/GCE electrode, the prepared sensor was activated in the presence of common interfering herbicides (CAR, MES and IRG) [61] and GLY analyte molecule and the obtained signal values were recorded (Figure 6). As expected, since a GLY molecule specific MIP based sensor was prepared, the prepared sensor showed more specific electrochemical properties towards the GLY molecule. The prepared MIP sensor was proven to recognize GLY 12 times more selectively than CAR, 16 times more selectively than MES, and 24 times more selectively than IRG (Table S1). Lastly, the values of the selectivity coefficient (k) and relative selectivity coefficient (k’) indicated that a high selectivity MIP sensor was prepared.

The stability test of the prepared MIP sensor was performed. For this purpose, the peak currents obtained by using a single MIP electrode in the presence of 10.0 nmol L^−1^ GLY for 8 weeks were measured (Figure S5). The close peak current values obtained over 8 weeks proved that the stability of MIP/Pd/Y_2_O_3_@BN/GCE electrode was high.

60 consecutive measurements were recorded by using one MIP/Pd/Y_2_O_3_@BN/GCE in the presence of 10.0 nmol L^−1^ GLY. After each measurement, the MIP electrode was washed with pure water. The relative standard deviation (RSD) value was 0.97% for these 60 current values, confirming the good repeatability of MIP/Pd/Y_2_O_3_@BN/GCE.

For the reproducibility of MIP/Pd/Y_2_O_3_@BN/GCE, 30 different MIP electrodes were prepared according to the electrode preparation procedure. A total of 30 different current values were recorded by interacting separately with 30 different electrodes in the presence of 10.0 nmol L^−1^ GLY. RSD of these current values was found to be 0.37%, confirming the good reproducibility of MIP/Pd/Y_2_O_3_@BN/GCE.

### 3.7. The Greenness Evaluation

The greenness profiles of MIP/Pd/Y_2_O_3_@BN/GCE were evaluated via Green Analytical Procedure Index (GAPI) and Analytical GREEnness (AGREE). GAPI pictograms with green, yellow, and red colors revealed all functions of MIP/Pd/Y_2_O_3_@BN/GCE. The result of AGREE from 0.0 to 1.0 was 0.83, showing the green method (Figure S6) and BAGI tools (Figure S7) demonstrated the high practicality assessment of MIP/Pd/Y_2_O_3_@BN/GCE.

## 4. Conclusions

Here, we developed a sensitive and specific detection method based on palladium@yttrium oxide@boronnitride nanocomposite and molecularly imprinting polymer for GLY assay. MIP/Pd/Y_2_O_3_@BN/GCE showed superior electrochemical properties for herbicide determination owing to the synergistic function of boronnitride, Y_2_O_3_ and palladium. The sensor accomplished a current of 14.04 µA, which was up to 8.02 times higher than that of NIP/Pd/Y_2_O_3_@BN/GCE electrode. Under optimal conditions, the limit of detection for this prepared MIP electrode was observed as 3.3 × 10^−10^ mol L^−1^ in range from 1.0 × 10^−9^ to 1.0 × 10^−8^ mol L^−1^ GLY. Additionally, this prepared MIP electrode revealed excellent selectivity, stability, repeatability, and reproducibility. MIP/Pd/Y_2_O_3_@BN/GCE also showed good performance in drinking water applications with recoveries of 99.85–100.22% for GLY determination. This study highlighted the high potential of Pd/Y_2_O_3_@BN nanocomposite as low-cost materials for herbicide assays in environment and food samples. Future experiments will focus on improving the long-term stability (exceeding 8 weeks).

## Figures and Tables

**Table 1:** The comparison of GLY imprinted Pd/Y_2_O_3_@BN/GCE’s analytical performance with the studied methods.

Method	Linear Range (M)	LOD (M)	Real Sample	Ref.
MIPNs	1.5 × 10^−2^–6.0 × 10^−3^	1.2 × 10^−2^	Orange juice	[62]
Fluorescence silicon nanoparticles (SiNPs)	8.9 × 10^−7^–8.9 × 10^−6^	1.8 × 10^−8^	Tap water and rice	[63]
CS/ZnO/SPR	0–6.0 × 10^−7^	8.0 × 10^−9^	-	[64]
CuAl-LDH/Gr NCs	3.0 × 10^−9^–1.2 × 10^−6^	1.0 × 10^−9^	Surface water	[65]
Liquid crystal (DOPG/Cu^2+^)	1.0 × 10^−9^–3.0 × 10^−7^	2.6 × 10^−10^	-	[66]
Electrochemical aptasensing: Differential pulse voltammetry	1.0 × 10^−9^–5.0 × 10^−6^	6.7 × 10^−10^	-	[67]
MIP/Pd/Y_2_O_3_@BN/GCE	1.0 × 10^−9^–1.0 × 10^−8^	3.3 × 10^−10^	Drinking water	This study

**Table 2:** Recovery results of GLY (*n* = 6).

MIP/Pd/Y_2_O_3_@BN/GCE				ICP/MS	
Sample	Added GLY (nmol L^−1^)	Found GLY (nmol L^−1^)	* Recovery (%)	Found GLY (nmol L^−1^)	* Recovery (%)
Drinking water	-	2.47 ± 0.06	-	2.46 ± 0.04	-
	2.00	4.48 ± 0.05	100.22 ± 0.01	4.46 ± 0.08	100.00 ± 0.06
	4.00	6.46 ± 0.02	99.85 ± 0.04	6.45 ± 0.05	99.85 ± 0.07
	6.00	8.47 ± 0.04	100.00 ± 0.02	8.47 ± 0.04	100.12 ± 0.03

* Recovery = Found GLY, nmol L^−1^/Real GLY, nmol L^−1^.

## Data Availability

The original contributions presented in the study are included in the article/Supplementary Materials. Further inquiries can be directed to the corresponding author.

## Supplementary Materials

The following supporting information can be downloaded at: https://www.mdpi.com/article/10.3390/foods15010007/s1, Figure S1. EDX MAPS spectrum of Pd/Y_2_O_3_@BN nanocomposite; Figure S2. XRD pattern of (A) hexagonal boronnitride material and (B) Pd/Y_2_O_3_@BN nanocomposite; Figure S3. SEM image of (A) MIP/Pd/Y_2_O_3_@BN/GCE and (B) NIP/Pd/Y_2_O_3_@BN/GCE; Figure S4. Effect of (A) pH, (B) mole ratio GLY to Py monomer, (C) elution time, (D) scan cycle on signals of SWVs (in presence of 10.0 nmol L^−1^ GLY) (*n* = 6); Figure S5. Stability test of MIP/Pd/Y_2_O_3_@BN/GCE including 10.0 nmol L^−1^ GLY by using SWV method (*n* = 6); Figure S6. Method greenness assessment tools pictograms; Figure S7. Practicality assessment of MIP/Pd/Y2O3@BN/GCE using BAGI tools (A web app version of BAGI tool is available at https://bagi-index.anvil.app); Table S1. k and k′ values of GLY imprinted electrodes (MIP/Pd/Y_2_O_3_@BN/GCE and NIP/Pd/Y_2_O_3_@BN/GCE) (*n* = 6).
