# Supplementary material for: Molecularly Imprinted Electrochemical Sensor Based on Palladium@Yttrium Oxide@Boronnitride Nanocomposite for Determination of Glyphosate Herbicide in Drinking Water Samples
Source: Foods. 2025 Dec 19;15(1):7. doi: 10.3390/foods15010007 (PMC12786010; doi:10.3390/foods15010007)
Supplement: Supplementary file 1 [file foods-15-00007-s001.zip › foods-4041307-supplementary.pdf]

Supplementary Data

For

Molecularly imprinted electrochemical sensor based on palladium@yttrium oxide@boronnitride nanocomposite for determination of glyphosate herbicide in drinking water samples

Bahar Bankoğlu Yola[1], Sena Bekerecioğlu[2], İlknur Polat[2], Ülkü Melike Alptekin[3], Necip Atar[4], Mehmet Lütfi Yola[5*]

[1]Department of Engineering Basic Sciences, Faculty of Engineering and Natural Sciences, Gaziantep Islam Science and Technology University, Gaziantep, 27260, Türkiye; bahar.bankogluyola@gibtu.edu.tr (B.B.Y)
[2]Department of Nutrition and Dietetics, Faculty of Health Sciences, Hasan Kalyoncu University, Gaziantep, 27010, Türkiye; sena.bekerecioglu@hku.edu.tr (S.B); ilknur.polat@hku.edu.tr (I.P)
[3]Department of Medical Services and Techniques, Dörtyol Vocational School of Health Services, Iskenderun Technical University, Hatay, 31200, Türkiye; ulku.alptekin@iste.edu.tr (U.M.A)
[4]Department of Chemical Engineering, Faculty of Engineering, Pamukkale University, Denizli, 20160, Türkiye; natar@pau.edu.tr (N.A)
[5]Department of Biology, Faculty of Science, Ankara University, Ankara, 06100, Türkiye

*Correspondence: mehmetlutfiyola@ankara.edu.tr; Tel.: +90-3122168600; Fax: +90-3122868900

2.2. Instrumentation

Scanning electron microscopy (SEM, ZEISS EVO 50 SEM, Tokyo, Japan), Transmission electron microscopy (TEM, JEOL 2100 TEM, Tokyo, Japan), PHI 5000 Versa Probe type x-ray photoelectron spectroscopy (XPS, Japan/USA) and Rigaku X-ray diffractometer (XRD, Germany) were used for the structural characterizations. The measurements of electrochemical impedance spectroscopy (EIS), square wave voltammetry (SWV) and cyclic voltammetry (CV) were done by using GAMRY Reference 600 workstation. The glassy carbon electrode (GCE) as working electrode was used with a geometric area of 0.073 cm^2, the reference electrode was a $Ag/AgCl/KCl_{(sat)}$ in aqueous media and the counter electrode was a Pt wire.

3.5. Sensitivity of MIP/Pd/Y_2O_3@BN/GCE electrode

$$LOQ = 10.0\ S / m$$

$$LOD = 3.3\ S / m$$

S: Standard deviation of the intercept and m*:* Slope of the regression line

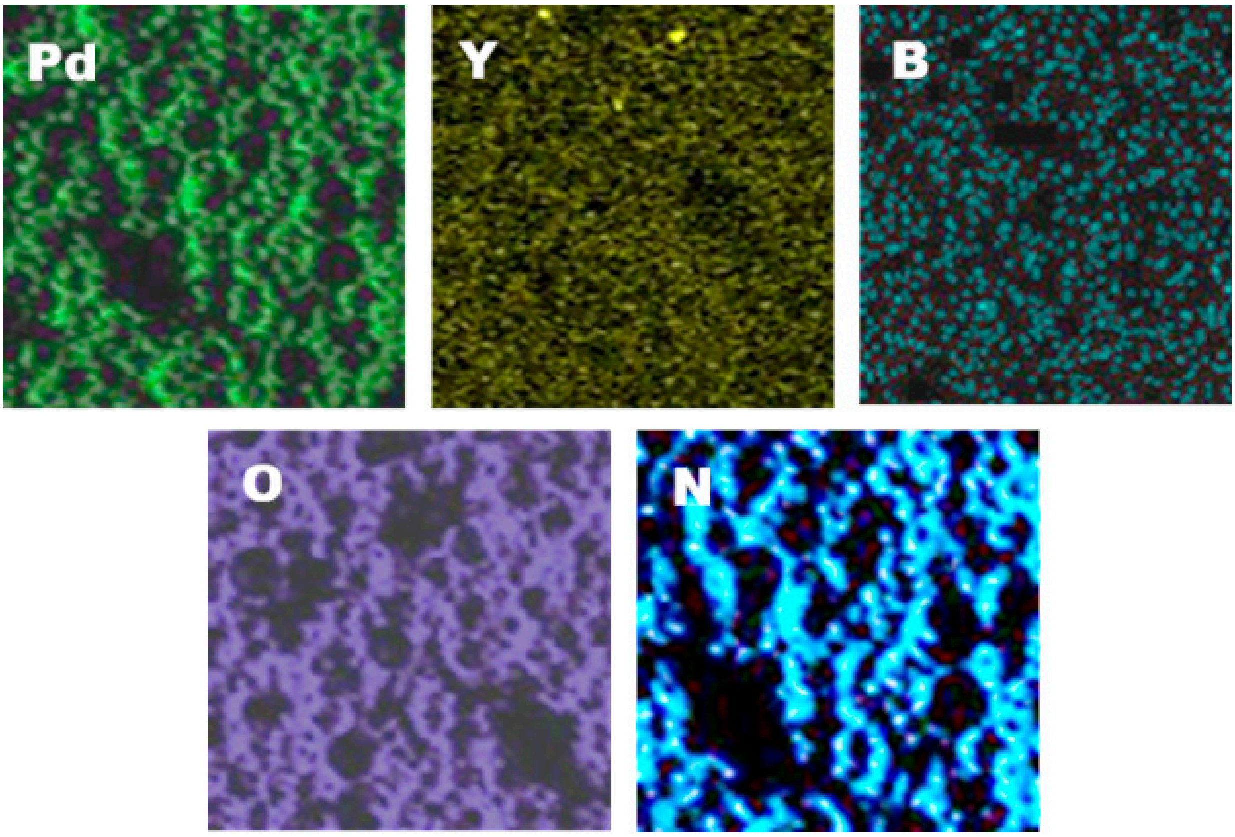

Figure S1. EDX MAPS spectrum of Pd/Y_2O_3@BN nanocomposite

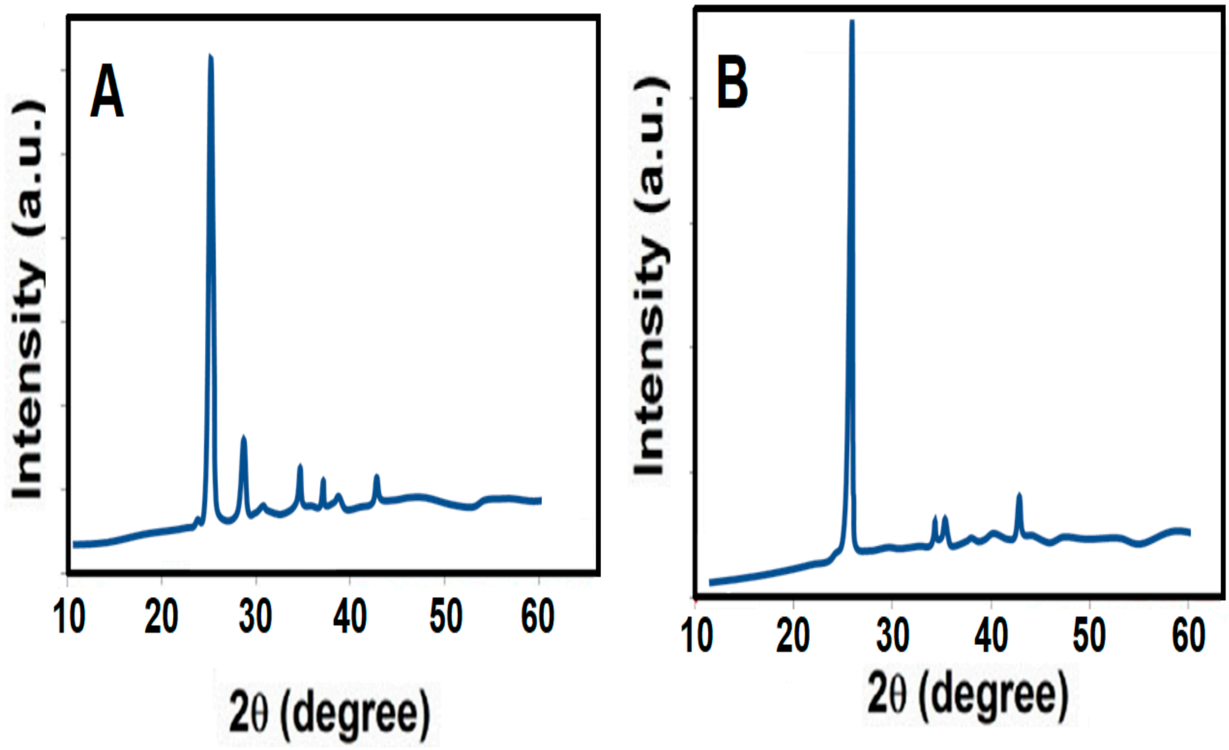

Figure S2. XRD pattern of (A) hexagonal boronnitride material and (B) Pd/Y_2O_3@BN nanocomposite

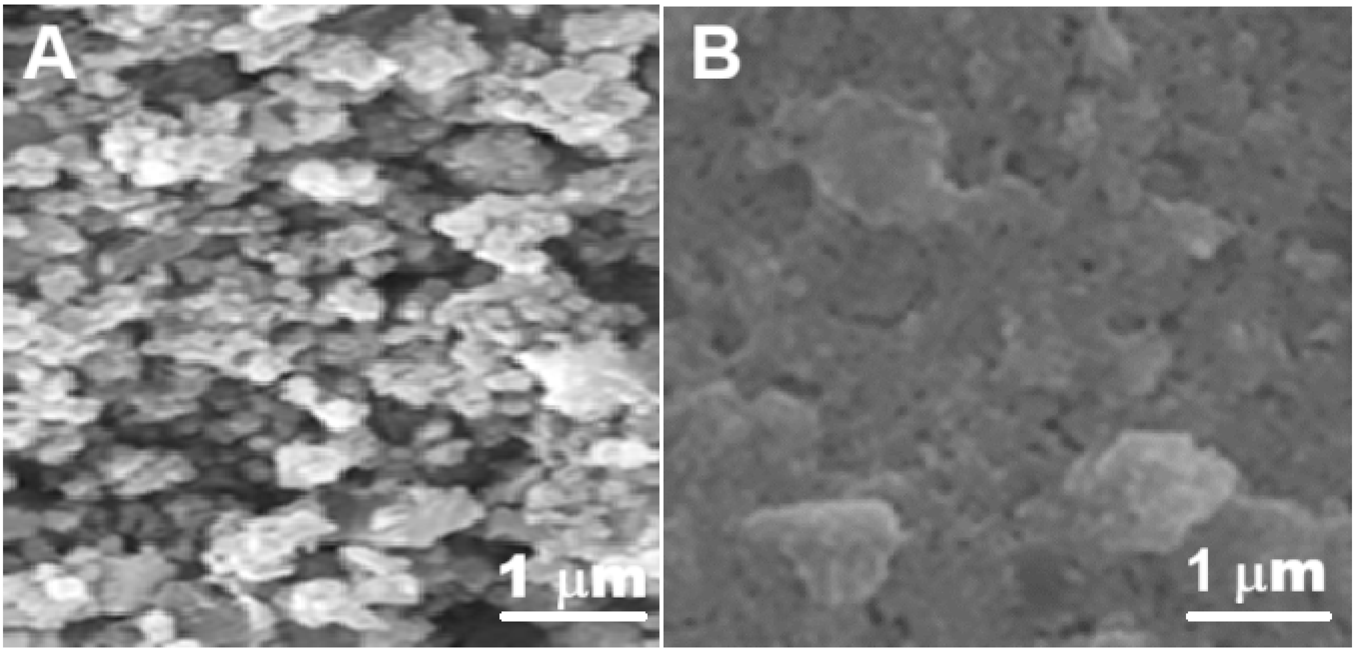

Figure S3. SEM image of (A) MIP/Pd/Y_2O_3@BN/GCE and (B) NIP/Pd/Y_2O_3@BN/GCE

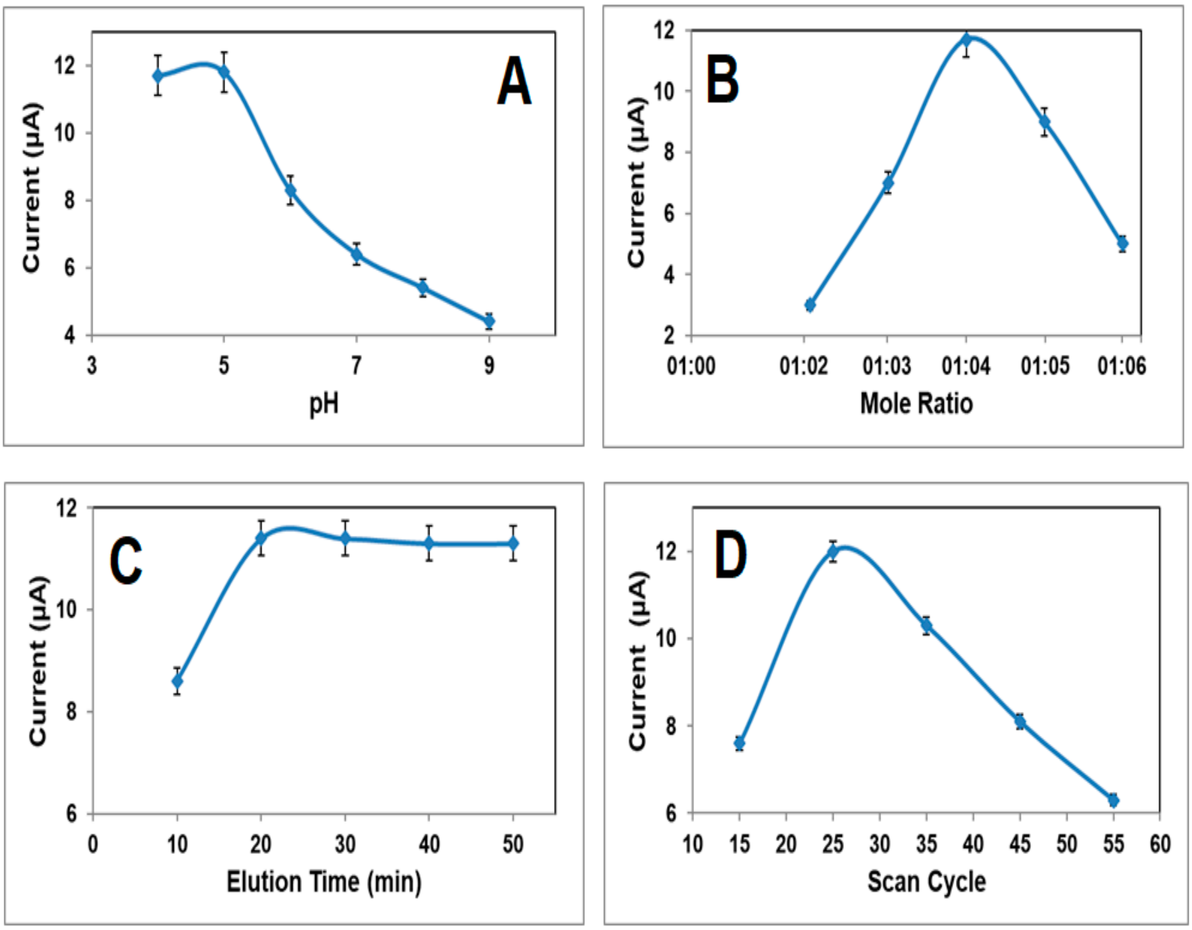

Figure S4. Effect of (A) pH, (B) mole ratio GLY to Py monomer, (C) elution time, (D) scan cycles on signals of SWVs (in presence of 10.0 nmol L^{-1} GLY) (n=6)

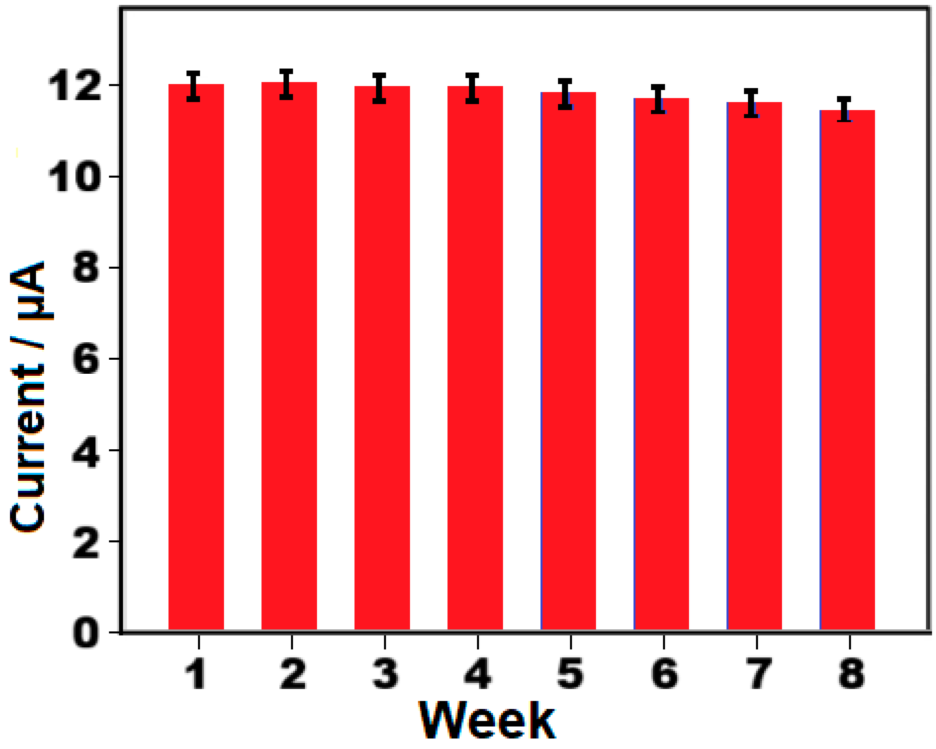

Figure S5. Stability test of MIP/Pd/Y_2O_3@BN/GCE including 10.0 nmol L^{-1} GLY by using SWV method (n=6)

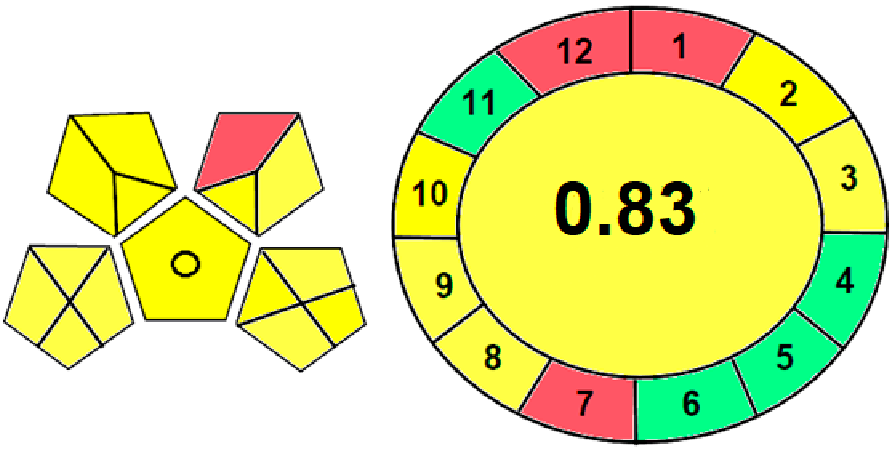

Figure S6. Method greenness assessment tools pictograms

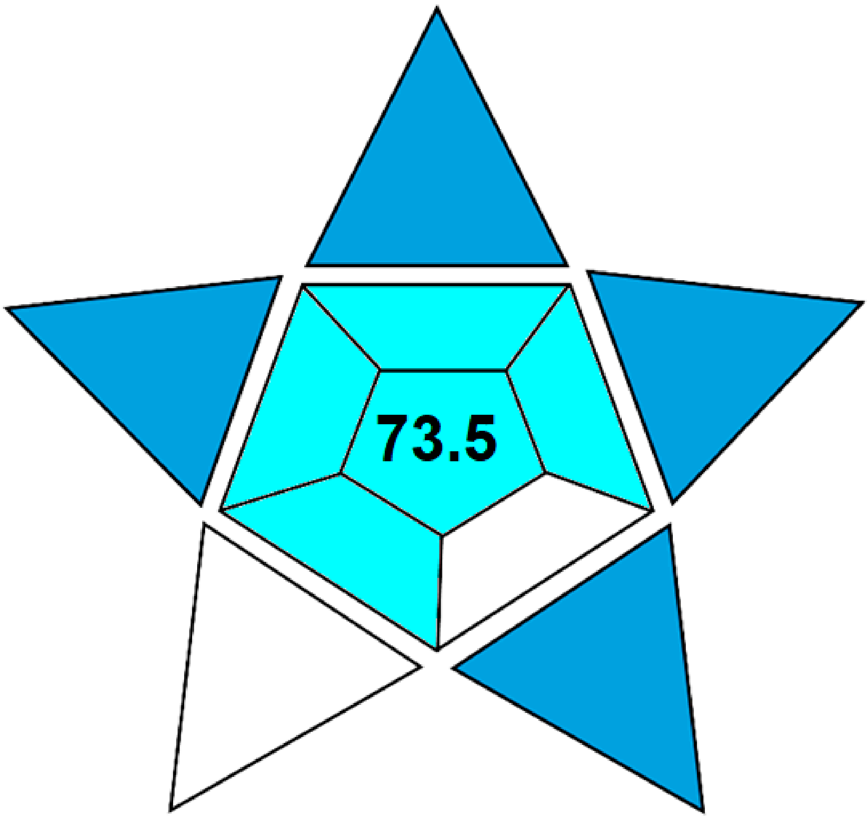

Figure S7. Practicality assessment of MIP/Pd/Y_2O_3@BN/GCE using BAGI tools

Table S1. k and k′ values of GLY imprinted electrodes (MIP/Pd/Y_2O_3@BN/GCE and NIP/Pd/Y_2O_3@BN/GCE) (n=6)

	**MIP**		**NIP**		
	**Δi (μA)**	**k**	**Δi (μA)**	**k**	**k′**
GLY	12.0 ± 0.01	-	1.50 ± 0.03	-	-
CAR	1.00 ± 0.04	12.0	1.00 ± 0.02	1.50	8.00
MES	0.75 ± 0.02	16.0	0.50 ± 0.01	3.00	5.33
IRG	0.50 ± 0.05	24.0	0.25 ± 0.01	6.00	4.00

Analyte concentrations: 10.0 nmol L^{-1} GLY, 100.0 nmol L^{-1} CAR, 100.0 nmol L^{-1} MES and 100.0 nmol L^{-1} IRG
$k = \Delta i_{GLY}/\Delta i_{\text{interfering chemical}}$ and $k' = k_{MIP}/k_{NIP}$
